# A promising hypothesis of c-KIT methylation/ expression paradox in c-KIT (+) squamous cell carcinoma of uterine cervix ----- CTCF transcriptional repressor regulates c-KIT proto-oncogene expression

**DOI:** 10.1186/s13000-015-0438-2

**Published:** 2015-11-25

**Authors:** Shih-Wen Chang, Wan-Ru Chao, Alexandra Ruan, Po-Hui Wang, Jau-Chen Lin, Chih-Ping Han

**Affiliations:** Department of Surgery, School of Medicine, Chung-Shan Medical University and Chung-Shan Medical University Hospital, Taichung, Taiwan; Department of Pathology, Chung-Shan Medical University and Chung-Shan Medical University Hospital, Taichung, Taiwan; Keck School of Medicine of the University of Southern California, Los Angeles, CA, USA; Department of Obstetrics and Gynecology, School of Medicine, Chung-Shan Medical University and Chung-Shan Medical University Hospital, Taichung, Taiwan; Department of Respiratory Therapy, Fu-Jen Catholic University, New Taipei, Taiwan

**Keywords:** Squamous cell carcinoma of uterine cervix, c-KIT proto-oncogene, CTCF repressor

## Abstract

We recently reported one interesting case showing mutation-free *c-KIT* proto-oncogene overexpression and paradoxical hypermethylation in 54 cases of primary squamous cell carcinoma of uterine cervix (SCC). However, its molecular mechanisms still remain unknown. We propose the hypothesis that increased methylation at the CpG islands on the promoter near the first exon region might interfere with the binding of CTCF repressor with *c-KIT* promoter that regulates *c-KIT* proto-oncogene expression in such case. Further studies focusing on the status of epigenetic modifications of mutation-free c-KIT (+) tumors are encouraged.

## Background

Epigenetic modifications may occur that result in changes in gene expression. Epigenomes represent an attractive therapeutic target. Current use of agents targeting epigenetic changes has become an interesting topic in cancer research. Additional evidence suggests that DNA methyltransferase inhibitors may serve as efficient chemo- and radiosensitizers in solid tumors [1]. Furthermore, tight association of DNA methylation and silencing of gene expression have been already established. Hypomethylation is the mechanism for ectopic oncogene activation whereas hypermethylation is the mechanism for tumor suppressor gene inactivation [2, 3]. In 54 cases of primary squamous cell carcinoma of uterine cervix (SCC) of uterine cervix, we recently demonstrated one case showing mutation-free, over-expression and paradoxical hypermethylation of the *c-KIT* proto-oncogene [4–6]. Within this afore-mentioned unusual case (no. 26), the DNA methylation ratio of the promoter near the first exon region in c-KIT (+) tumor area (26 T) was higher than that in adjacent c-KIT (−) non-tumor cervical epithelium (26 N). On the other hand, when comparing two cases with different c-KIT expression (no. 12 and no. 26), the DNA methylation ratio in the c-KIT (+) tumor area (26 T) was higher than that in another randomly selected c-KIT (−) tumor area (12 T) and adjacent c-KIT (−) non-tumor cervical epithelium (12 N) [4]. However, the molecular mechanisms for this difference still remain unknown.

The analysis of results from MethHC, a database of DNA methylation and mRNA expression profiles in human cancer showed that hyper-methylation in the promoter region of *c-KIT* proto-oncogene induced the down-regulation of gene expression in most cancer tissues such as colon adenocarcinoma. Conversely, the increased DNA methylation ratio in upstream 500 bps of promoter region enhanced the expression of *c-KIT* proto-oncogene in uterine corpus endometrial carcinoma [7, 8]. This phenomenon is similar to our previous findings in the unusual uterine cervix carcinoma case [4]. Intriguingly, this is counter to the current understanding that aberrant DNA methylation tends to silencing of genes.

Transcriptional repressor CTCF also known as 11-zinc finger protein or CCCTC-binding factor is a transcription factor in humans that is encoded by the CTCF gene [9]. Recent references demonstrated that absent or un-bound CTCF was associated with increased DNA methylation at a gene promoter in either normal or cancer cells [10, 11]. In addition, Lai et al. demonstrated that DNA methylation can activate the expression of CTCF-silenced oncogene BCL6 via blocking the binding of CTCF repressor to the first intron region [12]. Conversely, inhibition of DNA methyltransferase decreases BCL6 transcription due to the binding of CTCF to DNA in the methylation-sensitive region. Our previous studies and cumulative references suggested that increased methylation at the CpG islands, which are found on the promoter near the first exon region, might interfere with the binding of CTCF repressor with *c-KIT* promoter that regulates *c-KIT* proto-oncogene expression in such a case.

## Presentation of the hypothesis

**Fig. 1:**
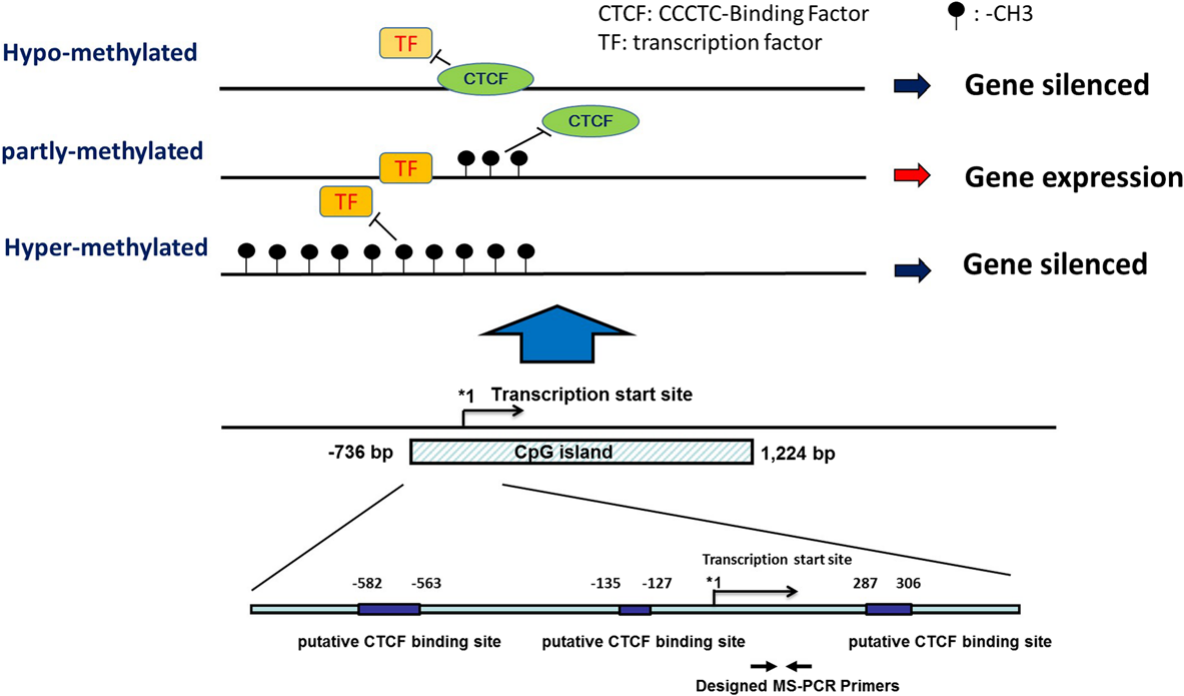
The analysis of the *c-KIT* proto-oncogene promoter region and the prediction of CTCF binding sites. The c-KIT proto-oncogene contains a long CpG island including promoter, first exon and first region ranging from −736 bp to 1224 bp. The CpG island region contains at least three putative transcriptional repressor CTCF binding sites predicted by the CTCFBSDB 2.0 database [13–15]. The methylation-specific (MS) PCR primer sets were designed to be located in the region between the first Exon and first intron by using MethPrimer software [16, 17]. The *blue box* indicates the putative CTCF binding sites. *1 indicates the transcription start site of *c-KIT* proto-oncogene

In this report, we propose a hypothesis for the “paradox” phenomenon between aberrant methylation and expression of *c-KIT* proto-oncogene in SCC uterine cervix. Increased methylation at the CpG islands of c-KIT promoter might interfere with the binding of certain repressor proteins such as CTCF transcriptional repressor (TR) that regulate c-KIT proto-oncogene expression. In general, CTCF binds to the CTCF-binding site on the promoter near the first exon region, as shown in Fig. 1. Subsequently, the hypo-methylation of the promoter region results in the silencing of the *c-KIT* proto-oncogene expression through interference with other transcription factors (TFs) to bind the promoter region. When CTCF-binding sites are partly methylated during tumorigenesis in uterine cervix, CTCF is unable to bind the methylated promoter region and loses the repressive function. Therefore, the TFs can smoothly bind to the promoter region and drive c-KIT proto-oncogene expression. In addition, when the CpG islands of the c-KIT promoter region are hyper-methylated, CTCF and TFs both are unable to bind to their binding sites and then the *c-KIT* proto-oncogene is silenced again (Fig. 1).

## Testing the hypothesis

1. To test this hypothesis, we must first sequence the methylation status of full length CpG islands of *c-KIT* proto-oncogene on promoter regions in several candidate cervical cancer cells by using bisulfide sequencing or pyrosequencing, and then correlate DNA methylation with the expression levels of *c-KIT* proto-oncogene in these cells. For our testing cell model, we will select the cells that are both hypermethylated at the CpG islands on the promoter near the first exon region and also express higher levels of *c-KIT* mRNA measured by quantitative RT-PCR.
2. Second, we will treat the cells with the DNA methyltransferase inhibitor 5-aza-2′-deoxycytidine (5-Aza-C), and test the change in expression level of the *c-KIT* proto-oncogene by quantitative RT-PCR. We hope to find that the expression of c-KIT proto-oncogene is related to the methylation of CpG islands, which are found on the promoter near the first exon region containing three putative CTCF-binding sites.
3. Third, we will analyze the amount of CTCF binding to the promoter region with three putative CTCF-binding sites by using a chromatin immunoprecipitation assay with specific primers to test cells treated with various concentration of 5-Aza-C. We will observe that the amount of CTCF binding to the promoter region increases with greater doses of 5-Aza-C. This will means that de-methylation on the promoter region enhances the binding activity of CTCF on the promoter region with three putative CTCF-binding sites.
4. Fourth, we will intensify or diminish the expression of CTCF and then measure the expression of *c-KIT* proto-oncogene to prove whether CTFC could directly bind to the promoter region to regulate the expression of *c-KIT* proto-oncogene.
5. Finally, we will assay the DNA methylation status and gene expression of *c-KIT* proto-oncogene in the large-scale clinical samples, and correlate the relationship between DNA methylation status and gene expression in SCC of the uterine cervix.

## Implications of the hypothesis

We propose the hypothesis that increased methylation at the CpG islands might interfere with the binding of the CTCF repressor with the *c-KIT* promoter which regulates *c-KIT* proto-oncogene expression in c-KIT (+) squamous cell carcinoma of uterine cervix. The paradoxical hypermethylation of the c-KIT promoter here suggests that therapeutic strategies using demethylating drugs could be effective against c-KIT (+) SCC of uterine cervix. Even though the low frequency (1/54 = 1.86 %) of mutation-free *c-KIT* proto-oncogene over-expression and aberrant methylation suggests that our result may not be representative of a larger population, further studies that focus on the status of epigenetic modifications of mutation-free c-KIT (+) tumors are still merited to clarify this point of view.

## Abbreviation

SCC: squamous cell carcinoma of uterine cervix

## References

[1] Gravina GL, Festuccia C, Marampon F, Popov VM, Pestell RG, Zani BM (2010). Biological rationale for the use of DNA methyltransferase inhibitors as new strategy for modulation of tumor response to chemotherapy and radiation. Mol Cancer.

[2] Wagner JR, Busche S, Ge B, Kwan T, Pastinen T, Blanchette M (2014). The relationship between DNA methylation, genetic and expression inter-individual variation in untransformed human fibroblasts. Genome Biol.

[3] Delpu Y, Cordelier P, Cho WC, Torrisani J (2013). DNA methylation and cancer diagnosis, Review. Int J Mol Sci.

[4] Chao WR, Lin WL, Chen CK, Han LM, Lin JC, Han CP (2015). Unusual c-KIT+ squamous cell carcinoma of the uterine cervix showing paradoxical hypermethylation of the c-KIT proto-oncogene. Eur J Obstet Gynecol Reprod Biol.

[5] Han CP, Lin WL, Wang PH, Yang SF, Lewis JS, Chen CK (2011). Overexpression of c-KIT (CD117) occurs infrequently in squamous cell carcinoma of the uterine cervix. Histopathology.

[6] Han CP, Chen CK, Lin CK, Wang PH, Chiang H (2011). Unusual c-KIT (+) squamous cell carcinoma of uterine cervix showing remarkable platelet-derived growth factor receptor, alpha subunit expression, but no activating mutation. Histopathology.

[7] Huang WY, Hsu SD, Huang HY, Sun YM, Chou CH, Weng SL (2015). MethHC: a database of DNA methylation and gene expression in human cancer. Nucleic Acids Res.

[8] MethHC: A database of DNA Methylation and gene expression in Human Cancer, June 2014: MethHC 1.0.3; http://MethHC.mbc.nctu.edu.tw. Assess 2 Nov 2015. 10.1093/nar/gku1151 PMC4383953 25398901

[9] Filippova GN, Fagerlie S, Klenova EM, Myers C, Dehner Y, Goodwin G (1996). An exceptionally conserved transcriptional repressor, CTCF, employs different combinations of zinc fingers to bind diverged promoter sequences of avian and mammalian c-myc oncogenes. Mol Cell Biol.

[10] Wang H, Maurano MT, Qu H, Varley KE, Gertz J, Pauli F (2012). Widespread plasticity in CTCF occupancy linked to DNA methylation. Genome Res.

[11] Dávalos-Salas M, Furlan-Magaril M, González-Buendía E, Valdes-Quezada C, Ayala-Ortega E, Recillas-Targa F (2011). Gain of DNA methylation is enhanced in the absence of CTCF at the human retinoblastoma gene promoter. BMC Cancer.

[12] Lai AY, Fatemi M, Dhasarathy A, Malone C, Sobol SE, Geigerman C (2010). DNA methylation prevents CTCF-mediated silencing of the oncogene BCL6 in B cell lymphomas. J Exp Med.

[13] CTCFBSDB 2.0: A database for CTCF binding sites and genome organization. http://insulatordb.uthsc.edu/. Assess at 2 Nov 2015.

[14] Ziebarth JD, Bhattacharya A, Cui Y (2013). CTCFBSDB 2.0: a database for CTCF-binding sites and genome organization. Nucleic Acids Res.

[15] Bao L, Zhou M, Cui Y (2008). CTCFBSDB: a CTCF binding site database for characterization of vertebrate genomic insulators. Nucleic Acids Res.

[16] MethPrimer. http://www.urogene.org/methprimer/. Assess at 2 Nov 2015.

[17] Li LC, Dahiya R (2002). MethPrimer: designing primers for methylation PCRs. Bioinformatics.

